# Correlating pre-operative MRI measurements of metatarsal Osteomyelitis with surgical clean margins reveals the need for a one centimeter resection margin

**DOI:** 10.1186/s13047-017-0222-5

**Published:** 2017-08-25

**Authors:** Brent Bernstein, Melody Stouder, Eric Bronfenbrenner, Steven Chen, David Anderson

**Affiliations:** 1 Department of Foot and Ankle Surgery, St. Luke’s University Health Network, 1736 W Hamilton St, Allentown, PA 18104 USA; 2 Department of Radiology, St. Luke’s University Health Network, 801 Ostrum St, Bethlehem, PA 18015 USA; 3 Department of Pathology, St. Luke’s University Health Network, 1736 W Hamilton St, Allentown, PA 18015 USA

**Keywords:** Osteomyelitis, Proximal margin, Clean margin, Metatarsal, MRI

## Abstract

**Background:**

Due to the high incidence of forefoot ulcerations with associated osteomyelitis, there has been an increased demand for partial ray amputations. In order to ensure complete removal of infected metatarsal bone, a clean margin amount is chosen based on the surgeon’s intraoperative visual subjective evaluation. The margin is resected and sent to pathology. Currently the literature shows positive proximal margin rates of 35–40%. The purpose of this study was to reduce the rate of positive proximal margins by effectively resecting all infected bone using pre-operative MRI measurements with an added resection margin.

**Methods:**

Twenty-four osteomyelitis positive metatarsals were included in this exploratory study. The distance of proximal osteomyelitic extension within the metatarsal was measured on MRI in centimeters. Intra-operatively, the partial ray amputation cut was determined by adding an extra 0.5 cm resection margin to the MRI measurement. At the study’s mid-point, bone histopathology revealed an increase in positive proximal margin rates-so the resection margin was increased to 1 cm. Descriptive outcomes included the mean distance of osteomyelitis propagation, proximal margin rates, as well as diagnostic statistics.

**Results:**

After removing the specimens with false positive MRI results, the study sample included 21 metatarsals positive for osteomyelitis. A 0.5 cm resection margin proximal to the osteomyelitis resulted in a 50% positive proximal margin rate. After increasing the resection margin to 1 cm, there was found to be an improved positive proximal margin rate of 9%. Based on MRI findings, the mean distance + standard deviation of osteomyelitis propagation along the metatarsal proximally was 1.81 cm + 0.74 cm. The metatarsal specimen was processed by pathology into multiple pieces and compared to MRI, resulting in MRI sensitivity of 67%, specificity of 74%, positive predictive value of 79%, and negative predictive value of 60%.

**Conclusions:**

By performing a 1 cm resection margin proximal to the metatarsal osteomyelitis the proximal margin rate was reduced to clinically meaningful levels. These preliminary findings support using a 1 cm resection margin when performing any form of metatarsal amputation, to reduce the risk of residual osteomyelitis post-operatively.

**Trial registration:**

St. Luke’s Hospital, IRB National Protocol ID SLHN2015–112. Date:1–13-16.

## Background

According to the Centers for Disease Control(CDC), 30.3 million Americans had diabetes in 2017, including 23.1 million diagnosed and 7.2 million undiagnosed [1]. An estimated 15% of patients with diabetes will develop a lower extremity ulcer during the course of their disease [2]. Osteomyelitis(OM) develops approximately 44–68% of the time when the diabetic patient is admitted to the hospital with an infection. Submetatarsal wounds are one of the common locations for skin breakdown and site of infection, thus considered a major contributor to metatarsal OM [3].

Due to the high rate of complications related to foot osteomyelitis, this study was created with the objective of reducing the positive proximal margin rates of metatarsal OM by utilizing pre-operative MRI measurements of OM.

Standard of care treatment of OM typically includes amputation with resection of the infected bone. The type of amputation is based on surgeon preference which most frequently includes a partial ray amputation. If three or more partial rays require amputation it is optimal to perform a transmetatarsal amputation in order to maintain biomechanical stability [4].

Regardless of the method of amputation, it is important that metatarsal excision is adequate and successful in resecting all infected bone. Typically, the bone is visually inspected intra-operatively by the surgeon and the amputation is established proximally until non-infected bone is reached. Visual confirmation of “healthy bleeding bone” is the typical benchmark for establishing successful resection of OM. However, this subjective evaluation can lead to surgical discrepancy [5]. The bone cut in the metatarsal must be placed proximal to the distribution of osteomyelitis; if not placed proximal to the OM it puts the patient at high risk for recurrence and spread of infection, ultimately increasing the odds of re-amputation. For example, a 10 year observational study of diabetic patients with amputation showed a re-amputation rate of 27.6% at 1 year, 48.3% at 3 years, and 60.7% at 5 years. These rates are alarming because re-amputation greatly increases morbidity and mortality [6].

In order to ensure adequate resection of osteomyelitic bone, a small portion of metatarsal bone from the most proximal cut can be sent to pathology and analyzed histologically-(otherwise known as a clean margin). If osteomyelitis is present in this specimen it is considered to be a “positive proximal margin”, with the result being interpreted as residual OM present in the foot. Treatment involves further bone resection or 4–6 weeks of IV antibiotics according to Infectious Disease Society of America guidelines [7].

A retrospective study by Kowalski et al. looked at 111 surgical margins resected for diabetic foot osteomyelitis and found that 35.14% of the specimens had OM positive margins [8]. Another retrospective observational study by Atway et al. showed a positive proximal margin rate of 40.7% in 27 patients who required toe, partial ray, and transmetatatarsal amputations. Eighty-one percent of the patients with positive proximal margins had poor outcomes compared to only 25% poor outcomes for the group with negative proximal margins [5].

Clearly, a 35–40% positive proximal margin rate resulting in residual foot osteomyelitis is unacceptable, especially when it contributes to increased morbidity and mortality. Adequate bone resection is necessary to remove all infection; however minimal resection is critical to limit biomechanical complications. Therefore, the extent of osteomyelitis propagation along the metatarsal should be determined pre-operatively in order to effectively eradicate all infected bone, while also minimizing excessive boney resection.

In this study, the overall goal was to decrease the rate of residual post-operative bone infection by improving the metatarsal rate of positive proximal margins. In order to fulfill this objective, the distance of OM was measured via MRI pre-operatively in order to create an adequate resection margin protocol. We are unaware of any study that has specifically used the MRI measurement of metatarsal OM in the operating room to obtain adequate bone resection.

## Methods

### Study design and participants

This prospective exploratory study was performed at a large teaching hospital network between the time period of January 2016 and May 2017 in order to evaluate the rate of residual osteomyelitis of the proximal metatarsal after operative resection. Nineteen patients (24 metatarsals) who were admitted with infected submetatarsal wounds with high suspicion of metatarsal OM were recruited for the study. Inclusion criteria were as follows: >18 years of age, infected forefoot wound with confirmed metatarsal OM on MRI, forefoot wound present prior to informed consent, and patient’s ability to provide own informed consent. Exclusion criteria were pregnancy, significant comorbidities affecting surgery, history of surgical intervention of the metatarsal in question, prior IV antibiotic treatment of the OM of the metatarsal in question, etiology of trauma or Charcot osteoarthropathy, recurrent osteomyelitis of the same metatarsal, and inability to have an MRI (e.g. defibrillators, pacemakers).

### Study procedure

Upon admission, patients received standard of care treatment, including a full infectious work up with IV antibiotics. Wound cultures were taken at bedside. The following wound characteristics were recorded: size of wound, presence of purulence, whether or not it probes to bone, and chronological time period of the wound. If the patient had multiple pedal wounds, only the submetatarsal wound with OM suspicion was recorded. Additional data that were collected included the anatomical metatarsal involved, co-morbidities, and the presence of co-existing peripheral arterial disease. A standard set of labs was ordered, which included a complete blood count, basic metabolic panel, HbA1c, and ESR. An MRI with and/or without IV gadolinium contrast was performed within the hospital network and read by staff radiologists. If OM was not present within the metatarsal, the patient was excluded from the study.

**Fig. 1:**
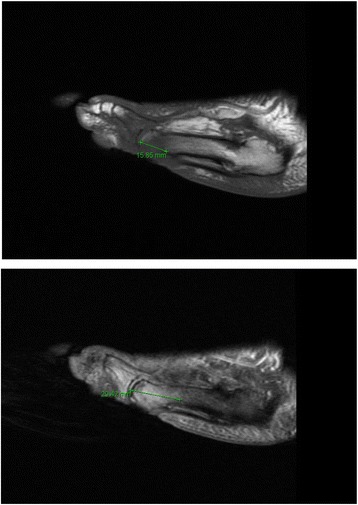
The top photograph displays a sagittal view of a T1 MRI image. The green line represents the distance of the proximal OM propagation. The bottom photograph displays a sagittal view of a T2 MRI image. The green line represents the distance of the proximal bone marrow edema propagation

If OM was confirmed on MRI, the radiologist measured the distance of proximal extension of OM along the metatarsal diaphysis in centimeters(cm) using specific protocol for determining the measurement distances. Total OM was measured from the distal tip of the metatarsal head to its most proximal location in the diaphysis. The radiology staff also measured the distance in centimeters of the bone marrow edema from the distal tip of the metatarsal head to its most proximal point within the diaphysis (Fig. 1). Osteomyelitis is considered as geographic and confluent with low intensity signals on T1-weighted images [9]. Therefore, the marrow fat on T1 that was completely replaced by a geographic area of decreased T1 signal was considered osteomyelitis and was measured in cm. The high intensity signals on T2-weighted images were measured, and this was considered bone marrow edema. If the decreased signals on T1-weighted images did not appear geographic or confluent, but instead presented as incomplete/hazy signals with a reticulated pattern they were labeled as bone marrow edema.

### Surgical intervention

**Fig. 2:**
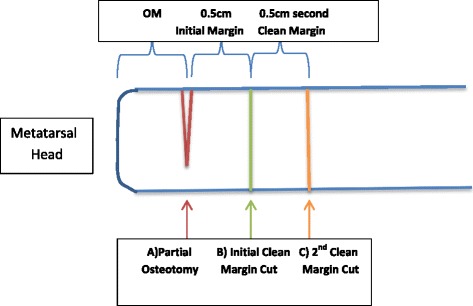
Surgical bone cuts within the metatarsal (A = partial osteotomy cut delineating where the OM terminates; B = the initial clean margin cut, 0.5 cm proximal to where the OM terminates; C = a separate clean margin cut, 0.5 cm proximal to (B).) According to the MRI the OM is located at level A. Modifying the procedure and adding an extra 0.5 cm clean resection margin overall results in 1 cm of bone resected proximal to the site of OM

After being medically cleared, the patient was taken to the operating room and the wound was adequately debrided and irrigated, removing all necrotic and infected tissue. All surgeries were performed by an experienced attending podiatrist and resident. The distance of OM that was reported previously by the radiologist was measured and mapped onto the metatarsal with a marker. A partial osteotomy cut was made at this location, where the osteomyelitis ends according to MRI. After copious irrigation, a new saw blade was used to make the through and through definitive bone cut 0.5 cm proximal to the partial osteotomy, thus providing a resection margin(i.e. the initial clean margin). This bone was removed as one piece and sent as histopathology. Halfway through the study, it was observed that an unacceptable number of patients had positive proximal margins. Therefore, the study team decided that in addition to following the above protocol, an extra 0.5 cm specimen of bone would be resected proximally and sent to histopathology separately as a second clean margin. Therefore, the overall results included in a total resection of an extra 1 cm “non-infected” bone, serving as the resection margin. Figure 2 depicts the locations of bone cuts. A small portion of bone from the metatarsal head was sent to microbiology for aerobic and anaerobic bone cultures. After which, the remaining bone specimens were sent immediately to pathology in a sterile preservative-filled container.

### Pathology procedure

**Fig. 3:**
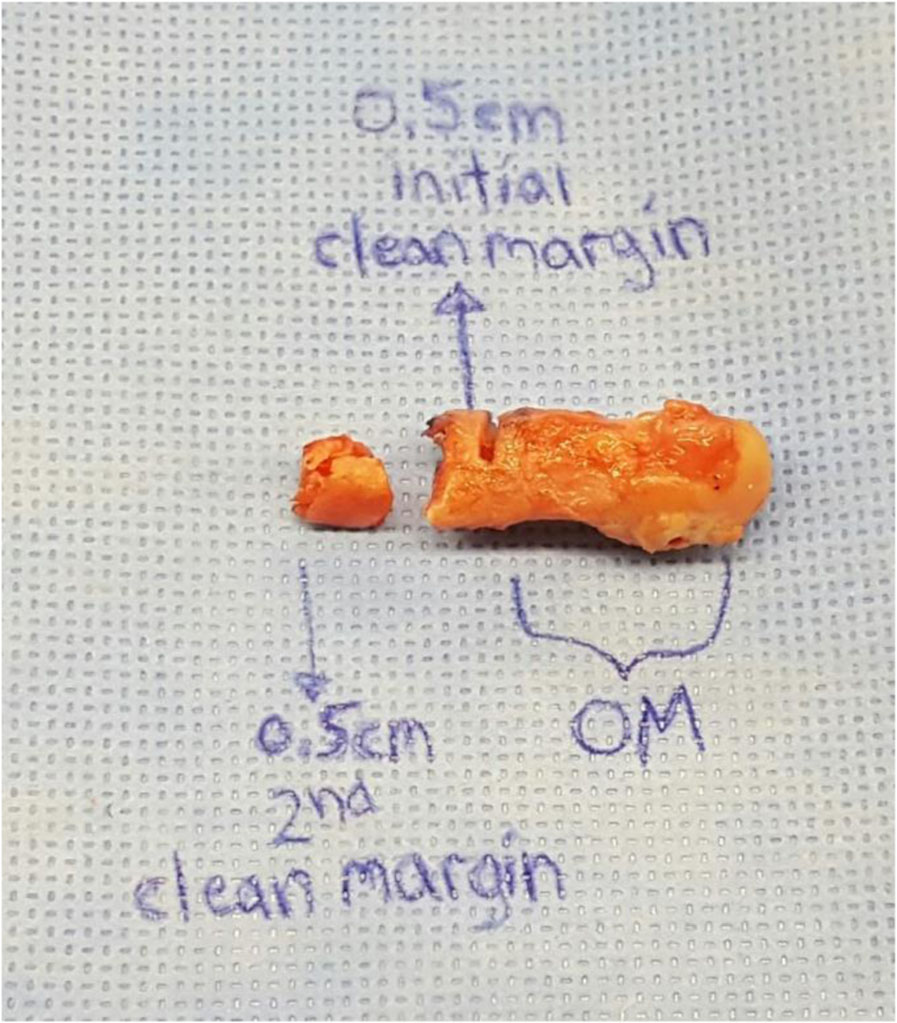
Surgical bone cuts within cadaver metatarsal

**Fig. 4:**
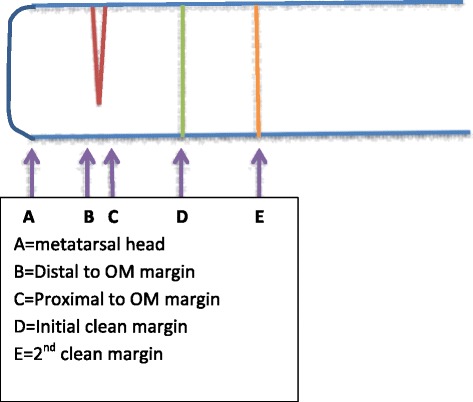
Locations of pathological specimens taken from the metatarsal

The pathology department processed the histological specimens according to a highly specific protocol. Representative samples of bone were taken from the metatarsal head, distal to the OM margin, proximal to the OM margin, the initial clean margin, and the 2nd clean margin (Fig. 3). All specimens were decalcified, placed in cassettes, and sent for processing into permanent slides. All histopathologic examinations were performed by one staff pathologist with a resident observer (Fig. 4).

Post-operatively, patients were considered to have completed their study involvement, and there were no study-related follow-up appointments. The literature has previously shown that positive proximal margins can increase the risk of post-operative complications, therefore the objective of this study was to reduce the rate of positive proximal margins; rather than to assess post-operative outcomes.

### Outcome measurement

The primary endpoint was to obtain the distance of proximal extension of metatarsal OM on MRI and resect 1 cm proximal to that measurement in order to reduce the rate of positive proximal margins. A secondary endpoint was to determine the accuracy of MRI OM compared to histological evaluation. Due to the exploratory nature of our study, we report descriptive outcomes including sensitivity, specificity, positive predictive value(PPV), and negative predictive value(NPV). The figures, graphs, and charts were created using Microsoft Word 2014.

## Results

A total of 24 metatarsal specimens(from 19 patients) were included in the study; basic demographics, wound characteristics, lab values, and imaging results were recorded (Table 1). The first ten metatarsal specimens within the study had a 0.5 cm resection margin of bone proximal to the osteomyelitis measurement. Five of these patients had a positive clean margin, resulting in a 50% rate of positive proximal margins (see Table 2). Because this rate was deemed unacceptable, additional bone was resected proximally. After adding 0.5 cm of bone resection (thus creating a total of 1 cm resection margin), the positive proximal margin rate reduced to 9.0%. In this second set of 14 patients, the MRI yielded a false positive in three cases, with the pathology being negative for OM. Therefore, these three specimens were removed from consideration, resulting in 11 patients with confirmed metatarsal OM who had a full 1 cm resection margin. Only one of the 11 patients had a positive proximal margin, resulting in an overall 9.0% positive proximal margin rate (Table 1). There were no false positive findings of OM on MRI for the first set of 13 patients (those without the 2nd clean margin).

**Table 1:** Participant Characteristics, Clinical Findings, and Demographics

Characteristics	Total (n) (%)
Size of wound: *n* = 19	
< 1 cm	5 26.3
1–5 cm	13 68.4
> 5 cm	1 5.3
Purulence: *n* = 19	
Yes	3 15.8
No	16 84.2
Probe to bone: *n* = 19	
Yes	12 63.2
No	7 36.8
Chronicity of Wound: *n* = 19	
Unsure	6 31.6
< 2 weeks	1 5.3
> 2 weeks	12 63.2
Anatomical Distribution of Metatarsal OM: *n* = 24	
1	1 4.1
2	4 16.7
3	3 12.5
4	3 12.5
5	13 54.2
Comorbidities: *n* = 19	
Diabetes	14 73.7
Hypertension	14 73.7
Peripheral arterial disease	7 36.8
Cardiovascular disease	5 20.8
Renal insufficiency	3 15.8
Liver Cirrhosis	3 15.8
Findings on Xray or MRI: *n* = 19	
Subcutaneous emphysema	1 5.3
Abscess	1 5.3
WBC: *n* = 19	
< 10	8 42.1
Ten to 14	6 31.6
> 14	5 26.3
ESR: *n* = 19	
< 60	11 57.9
60–90	5 26.3
> 90	3 15.8
A1c: *n* = 19	
< 6.5	11 57.9
Six.5 to nine	6 31.6
> 9	7 36.8

**Table 2:** Positive proximal margin rates for specimens with 0.5 cm and 1 cm resection margins

	No. of specimens	Negative Margin	Positive Margin	+ Margin Rate
0.5 cm resection margin	10 (specimens with FP = 0)	5	5	50.0%
1 cm resection margin	14–3(specimens with FP) = 11	10	1	9.0%

**Fig. 5:**
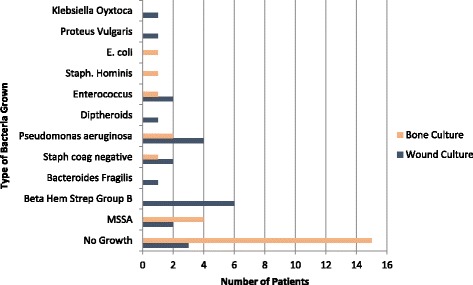
Bacterial growth from wound cultures and corresponding bone cultures

For each patient, a wound culture and bone culture was obtained. Figure 5 displays the types of bacteria grown from each wound and bone culture.

**Fig. 6:**
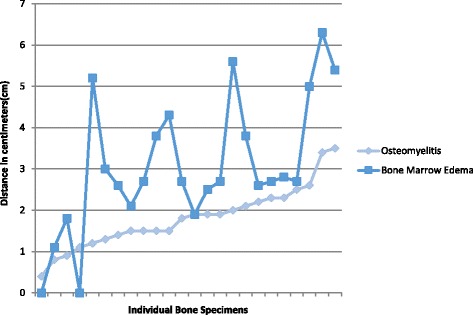
Distance(cm) of OM and bone marrow edema propagation along the metatarsal according to MRI

According to MRI results, the osteomyelitis traveled proximally an average of 1.81 cm from the metatarsal head. The bone marrow edema traveled proximally an average of 3.05 cm from the tip of the metatarsal head (Table 3). Figure 6 displays the distance(cm) of osteomyelitis and bone marrow edema for every specimen recorded.

**Table 3:** OM and bone marrow edema measurements on MRI

MRI Measurements	Mean	Standard Deviation (CI = 95%)	Range
Distance of OM	1.81	0.74	0.4–3.5
Distance of BME	3.05	1.63	0–5.4 cm

**Fig. 7:**
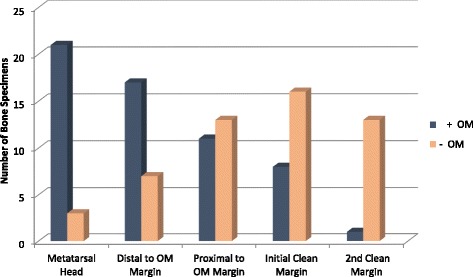
Number of OM positive versus OM negative pathological specimens

Once the specimens were received by pathology, bone samples were resected from the metatarsal head, distal to the OM margin, proximal to the OM margin, the initial clean margin, and the 2nd clean margin. Figure 7 displays each specimen taken from the bone and shows whether or not it was positive for OM.

**Fig. 8:**
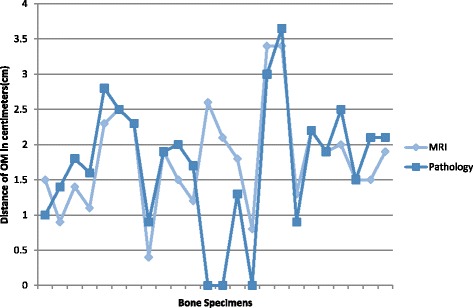
Distance(cm) of OM propagation observed on MRI versus pathology for each metatarsal

After all bone specimens were evaluated by a pathologist, the histological distance of OM proximal propagation was approximated. Figure 8 displays the distance of OM on MRI compared to the approximate distance of OM determined by pathology. In 50.0% of cases, pathology results revealed that the distance of OM was greater than the distance the MRI had predicted. In 16.7% of cases, pathology results revealed that the distance of OM was less than the distance the MRI had predicted. In 12.5% of cases the MRI was considered a false positive- while the MRI and Pathology distances of OM were the same in 25.0% of cases.

For MRI findings, the true positive (TP rate) was defined as a positive MRI and a positive biopsy; the false positive(FP) rate was defined as a positive MRI and negative biopsy; the true negative (TN) rate was defined as a negative MRI and a negative biopsy; and the false negative (FN) rate was defined as negative MRI and a positive biopsy (Table 4). The specimens labeled as metatarsal head and distal to OM margin should be positive for OM, according to the MRI findings. Therefore, all outcomes should be considered a true positive. Table 5 reveals that this was not the case when compared to pathology results. The specimens labeled as proximal to OM margin, initial clean margin, and second clean margin should all be negative for OM, according to the MRI findings. All of these specimens should be considered true negatives; however, Table 6 revealed otherwise according to pathology results. Table 7 lists the sensitivity, specificity, PPV, and NPV of the MRI findings. The second clean margin category was not included in these calculations, as only 14 specimens within the study actually had a second clean margin resected.

**Table 4:** MRI results versus pathology results

		Pathology	
MRI		Positive	Negative
	Positive	TP	FP
	Negative	FN	TN

**Table 5:** Metatarsal Head and Distal to OM Margin Specimens

	True positive	False positive
Metatarsal Head	21	3
Distal to OM Margin	17	7

**Table 6:** Proximal to OM margin, initial clean margin, second clean margin

	True Negative	False Negative
Proximal to OM Margin	13	11
Initial clean margin	16	8
2nd Clean Margin	13	1

**Table 7:** Sensitivity, specficity, PPV, and NPV of MRI

	Sensitivity	Specificity	PPV	NPV
MRI	67.0%	74.0%	79.0%	60.0%

## Discussion

Findings from this study indicate that resecting 1 cm of bone proximal to the metatarsal OM significantly decreased the positive proximal margin rate, therefore increasing the odds of successfully removing all osteomyelitic bone. Resecting only 0.5 cm of bone proximal to the level of osteomyelitis resulted in an increased positive proximal margin rate(50%). Based on these findings, we suggest making the definitive metatarsal amputation cut at least 1 cm proximal to the level of OM measured on MRI.

When evaluating anatomical distribution of metatarsal osteomyelitis, it was clearly evident that the 5th metatatarsal was the most common metatarsal infected, involving 54.2% of the metatarsals (Table 2). A retrospective review of 130 forefoot wounds showed that the two most common locations of wound formation included adjacent to the 5th metatarsal head at a rate of 19% and adjacent to the 1st metatarsal head(14%). Seventy-three percent of the wounds were infected and the 5th and 1st metatarsals had the highest rates of OM [10].

Sixty-two and a half percent of the final bone culture results included no growth of bacteria (Fig. 2). All patients were started on IV antibiotics upon admission so this is to be expected. A study by Asten et al. evaluated the types of bacteria involved in diabetic foot osteomyelitis by comparing a 16srRNA sequencing approach to the standard culture techniques. They took 34 bone samples and found that all specimens that had a negative bone culture ended up containing Staph spp. when sequenced with 16srRNA. Their explanation for this is that when antibiotics are administered, it can render the pathogens/bacteria non viable. Therefore the advantage to the 16srRNA sequencing was its ability to detect the bacteria even after the antibiotics rendered them inactive. In our study all of the patients received IV antibiotics upon admission and standard bone cultures were obtained, thus explaining the high percentage of bone cultures with no growth [11]. Only 28.6% of the initial wound cultures grew similar bacteria as the intra-operative bone cultures. In a prospective analytic study by Elamurugan et al., they found that out of 134 bone biopsy specimens, the bone bacterial pathogen was only identified in the corresponding swab culture 38.2% of the time [12].

Out of the entire study, there were six metatarsal bones that had a positive proximal margin, and in four of these patients the wounds had grown a component of Beta-hemolytic strep group B. Seng et al. retrospectively reviewed 93 cases of streptococcal bone and joint infections. They discovered a high rate of unfavorable clinical outcomes and amputations (17%) despite the low rate of antimicrobial resistance. The authors discuss the fact that there have been many developments in treatment protocols for staph., however no similar approaches exist for streptococcal bone and joint infections [13]. These findings suggest that even when strep is susceptible to an antibiotic, it can still have a high rate of pathogenicity. Strep infections can move quickly because of their genes which allow production of extracellular enzymes, streptokinases, cytolysins, streptolysins, capsules, and M proteins [14]. MRI as an imaging modality can detect OM as early as 3–5 days after the onset of infection [15, 16]. But it could be possible that if the bacteria is able to propagate quicker through the bone, the MRI may not detect the accurate distance of OM secondary to the delayed detection. For this reason, one should consider a more aggressive bone resection in the operating room if the original wound culture grows Beta-hemolytic strep group B.

Many studies have shown that MRI is the most accurate imaging modality when determining the extent of osteomyelitis. Magnetic resonance imaging has recently demonstrated high sensitivity(90–100%) and specificity(80–83%) in the detection and diagnosis of osteomyelitis in diabetic foot ulcers which is better than x-ray, ultrasound, or bone scans [17–19]. These sensitivities and specificities are higher than what was calculated in our study. In this study, the MRI was not globally detecting or ruling out the presence of OM in the metatarsal. We specifically had radiologists measure the exact distance in centimeters, and this isn’t always clear cut. In certain scenarios it can be easy to distinguish the OM measurement by visibly measuring the decreased signal intensity on T1-weight images. The overlap of the decreased signal on T1 and the increased signal of T2 is what gives us our diagnosis of OM [20]. The non-overlapping increased signal intensities on T2-weight images are considered bone marrow edema. The radiological terms for the decreased signal on T1 would be described as geographic and confluent. However there are situations in which the T1 signal in the marrow appears to be hazy, with a reticulated pattern, which is more indicative of bone marrow edema [21]. A study by Johnson et al. showed that when the bone edema was subcortical or had a hazy reticulated pattern, none of the cases were osteomyelitis [22, 23]. Therefore, when you start at the origin site of the bone infection(aka the metatarsal head) you expect to see decreased T1 signal with complete replacement of the marrow. However, as you move proximally down the diaphysis there is a gradual transition to bone marrow edema. As this occurs, the T1 signal begins to appear more reticulated and hazy. It can be difficult to distinguish an exact cut off line where the OM ends and the bone marrow edema begins. In our study we attempted to do so by using the MRI measurements. The pathological specimens were then histologically analyzed at several different locations within the metatarsal. This process therefore likely contributed to the lower sensitivity and specificity values of MRI when detecting OM.

Out of 24 bone specimens, three of them were read as false positives on MRI, as pathology concluded a complete absence of OM. There are many studies in the literature that discuss the complications of inacurrately diagnosing OM as bone marrow edema and vice versa. A study involving 12 diabetic patients looked at the efficacy of MRI evaluating the presence of OM and distinguishing it from bone marrow edema. All bony specimens with OM on MRI were confirmed by histopathologic examination with a 100% accuracy. However, 29 bone specimens were diagnosed with bone marrow edema by MRI and only 23 were histopathologically confirmed as much. The remaining six pieces were positive for OM, creating a 79.3% accuracy rate for diagnosing bone marrow edema by MRI [24]. Craig et al. performed a study where 18 of 57 bones that were analyzed demonstrated increased signal intensity by MRI, but only marrow edema at pathology [25].

Although MRI has been described as the gold standard imaging modality for detecting OM, there are still many discrepancies which can occur. This is why it is crucial to obtain an adequate amount of viable bone proximally(which serves as a resection margin) in order to ensure that the amputation is performed proximal to the OM. A prospective study by Simpson et al. placed patients with osteomyelitis into three groups: 1)bony resection with a 5 mm or more margin, 2)resection/clearance margin less than 5 mm, 3)de-bulking of infection with no margin. Zero patients in group 1 had recurrence, 28% had recurrent infection in group 2, and all patients had recurrence in group 3 [26]. Simpson’s study showed that creating an adequate resection margin can help ensure complete removal of osteomyelitis. Our protocol originally included a 0.5 cm resection margin, however due to suboptimal results it was decided the margin should be increased to 1 cm. After modifying the protocol, it was observed that the positive proximal margin rate drastically decreased by 82% (from 50.0% positive proximal margin rate to 9.0%).

Of the 24 bone specimens, six were considered to have a positive proximal margin; however, only three these were considered active OM by the pathologist. The other three specimens were described as resolving, chronic, and/or inactive. In order to keep our protocol straightforward and not skew the data, we decided to define all of them as + for OM. All of the pathological slides at our institution were read by one individual pathologist so there were no discrepancies or disagreements on the final diagnosis of OM or lack there of. Under histopathological evaluation, acute OM shows fragmented bony trabeculae, with neutrophils infiltrating the bone. Chronic OM has spongy osseous tissue, with the medullary space containing fibrosing granulation tissue with infiltrating macrophages, lymphocytes, plasma cells, and very few neutrophils [27]. The presence of chronic OM can be difficult to define, especially when deciphering if it is beginning or resolving. This can contribute to conflicting pathological assessment. A retrospective review by Meyr et al. reviewed 39 bone tissue specimens and had four different pathologists evaluate the specimens. There was complete agreement between all four pathologists only 33% of the time when it came to diagnosis of the bone. These results reveal that there is a need for a more comprehensive diagnostic protocol for OM [28].

The average distance the OM propagated along the metatarsal proximally, was 1.81 cm. Adding an adequate resection margin of 1 cm would lead to resection of metatarsal bone measuring 2.81 cm. There can be several scenarios where an urgent amputation is required (such as gas gangrene/abscess) or the patient may not be able to have an MRI. In these situations one might consider resecting at least 3 cm of bone, in hopes of increasing the odds of a surgical cure. This could potentially be used as an adequate reference standard in the operating room, especially when there is difficulty distinguishing viable bone from infected bone.

The present study had several important limitations. First, only 11 metatarsal specimens were included in the 1 cm resection margin group (after excluding the false positive MRI readings). This is a limited number of bone specimens and these results may not be representative of our entire institution or other institutions. Therefore it would be beneficial to perform a larger study that maintains our surgical protocol. Second, this study was performed at a single institution with only one pathologist interpreting the histopathological specimens. As discussed above, pathologists at times can have different view points on the diagnosis of OM. Therefore, our pathology results may have been interpreted differently at another institution. Third, the MRIs were interpreted by several different radiologists within our radiology department. Despite our strict protocol for measuring the MRI OM, there may have been subjective variations within the OM measurements.

## Conclusions

In conclusion, after the protocol adjustment was confirmed and a total of 1 cm of bone was resected proximal to the level of OM, the positive proximal margin rate decreased significantly to 9.0%. This a great improvement when compared to the rates found in the literature(35–40%). Before proceeding with a partial ray amputation we recommend having the distance of OM measured on MRI, then adding an extra 1 cm resection margin to decrease the incidence of residual bone infection post-operatively. Resecting inadequate bone intra-operatively can lead to increased co-morbidities for the patient. However, overzealous resection of bone could lead to biomechanical complications or transfer lesions. We believe that taking an extra 1 cm of bone proximally creates a fine balance between the two. Overall, we feel that our protocol has merit as it allows for a more precise and objective selection of margin resection with very low recurrence rates. Due to the small sample size of the study, it would be beneficial to have a second study with a larger population size that follows our protocol.

## Abbreviations

BME: Bone marrow edema
cm: centimeters
MRI: Magnetic resonance imaging
OM: Osteomyelitis
